# “Go for Gold!” or “just for fun”?—competitions at Special Olympics Unified Sports® at the National Games 2022

**DOI:** 10.3389/fspor.2025.1608690

**Published:** 2025-07-15

**Authors:** Steffen Greve, Tjorven Marie Göb, Jessica Süßenbach

**Affiliations:** ^1^ Department of Teaching Competence in Sport, Institute for Sports Science, Humboldt-University of Berlin, Berlin, Germany; ^2^ Unit for Physical Education and Sports Didactics, Institute for Physical Activity, Sports and Health, Leuphana University Lüneburg, Lüneburg, Germany

**Keywords:** participation, disabilities, competitive sport, handball, basketball

## Abstract

Special Olympics Unified Sports® is a sports program in which individuals with and without intellectual disabilities train and compete together on the same team. This study, designed and conducted in accordance with the Grounded Theory Methodology (GTM), investigates how individuals with and without intellectual disabilities co-experience and negotiate competitive dynamics in Unified Sports® basketball and handball. The study examined the success factors and challenges within this context. Based on 41 interviews players (14 athletes and 27 partners) conducted during the 2022 Special Olympics National Games in Berlin, the findings reveal pronounced hierarchical structures along the lines of disability. Partners often adopt a reserved, supportive role, intentionally holding back their own performance in order to place athletes at the center of gameplay. This approach is also evident in interactions with opposing teams, aiming to create a balanced and sporting experience for people with and without disabilities. Such practices challenge conventional notions of athletic success and point to an alternative logic of competition—one that prioritizes joyful social interaction over winning. At the same time, these support strategies carry the risk of fostering paternalistic dynamics that may limit the athletes' autonomy. Grounded in the methodology of Grounded Theory, the study is being extended through additional data collection at national and international competitions. The goal is to develop a differentiated system of categories and to contribute to a deeper understanding of competition in Unified Sports®.

## 1 Introduction

In Special Olympics Unified Sports®, individuals with and without disabilities compete both alongside and against one another in structured athletic settings. This constellation presents a notable complexity. At its core, competitive sport is oriented toward outperforming and ultimately defeating one's opponent. In contrast, social interactions between individuals with and without disabilities are typically characterized by support and assistance offered by the non-disabled toward their peers with disabilities. This inherent tension raises important questions about the nature of competition in Unified Sports®.

There is limited scientific knowledge regarding the context described. Greve et al. (1) identified hierarchical structures between individuals with and without disabilities, which were related to the disabilities. Most research on Unified Sports® or similar settings where people with and without disabilities participate in sports has focused on social interaction and the participation of people with disabilities (2–5). It has been repeatedly demonstrated that people without disabilities often hold back during the game and assume a supportive role for those with disabilities (3, 6). Such behaviors are inconsistent with the regulations established by Special Olympics for the competitive approach of Unified Sports®. This raises the central question of how Unified Sports® participants manage and experience this situation, in which winning and losing create excitement and emotions. This study addresses a critical gap in the literature by exploring competitive dynamics in Unified Sports® from the perspective of both athletes and partners, offering new insights into how Unified Sports® practices reshape traditional sport paradigms. This study seeks to gain an in-depth understanding of how competition is negotiated and perceived in Unified Sports®, with particular attention to the success factors and challenges that emerge from participants' experiences.

### 1.1 Special Olympics Unified Sports®

Special Olympics Unified Sports® is a program designed to bring together individuals with and without intellectual disabilities to train and compete on the same team. Within this framework, individuals with intellectual disabilities are referred to as *athletes*, while those without intellectual disabilities are termed *partners*. Special Olympics distinguishes between three distinct models within the Unified Sports® framework (7). The Recreational Model prioritizes joint physical activity regardless of participants' skill levels and excludes formal competition. Its primary objective is to foster inclusive and enjoyable sports experiences for all participants. The Player Development Model emphasizes mutual learning between participants with varying levels of ability, aiming to enhance sport-specific skills through peer support. While this model allows for competitive participation, it is limited to national-level events. The Competitive Model, which is the focus of the present study, is oriented toward structured training and participation in both national and international Special Olympics competitions. Within this model, it is recommended that athletes and partners are of similar age and possess comparable sport-specific skills to ensure equitable performance levels (7). This investigation centers on the application of the Competitive Model in the sports of handball and basketball. Specific regulations govern team composition in these sports: in handball, four athletes and three partners must be on the field simultaneously, with an additional stipulation in Germany requiring the goalkeeper to be an athlete (18). In basketball, three athletes and two partners play together on the court.

To ensure meaningful participation for all players, Unified Sports® is guided by the *Principle of Meaningful Involvement* (8). This principle mandates that all team members contribute to the team's success by actively engaging in play, utilizing their individual strengths, and assuming key roles within the game. Dominance by individual players or exclusion of others is explicitly prohibited. Temporary moderation of individual abilities may be encouraged to maintain team balance; however, such adjustments should not be excessive or prolonged. Violations of this principle include players acting as de facto coaches on the field or inconsistent participation in regular training sessions (8). With regard to the role of partners, Special Olympics Germany (2021) provides explicit guidelines. Partners are expected to serve as role models and to actively involve athletes in gameplay. These guidelines establish a functional asymmetry between athletes and partners, as partners are implicitly assigned a higher level of performance and a supporting role. This structural distinction may significantly shape participants' experiences and perceptions within the Unified Sports® context.

### 1.2 Competition structure within special olympics

Special Olympics competitions are held at local, regional, national, and international levels (9). These events are governed by the official Special Olympics rules, which are generally based on the regulations of international and national sport federations. However, modifications may be implemented to accommodate the specific needs of athletes with intellectual disabilities. To ensure fair and equitable competition, participants are grouped into *competition divisions* based on ability. This is achieved through a pre-competition classification process known as *divisioning* (7). The purpose of divisioning is to create performance-homogeneous groups that facilitate fair and engaging competition experiences for all participants.

In the sport of handball, divisioning is conducted according to the Swiss system, in which a ranking is established based on match outcomes. To further ensure fairness, the *Maximum Effort Violation* rule is applied. This regulation prohibits athletes or teams from intentionally underperforming during the divisioning process in order to be placed in a lower division. Such actions are considered violations because rankings within each division are awarded at the end of the tournament, including the allocation of medals.

In addition, the *Criteria for Advancement to Higher Level Competition* govern eligibility for participation at subsequent competition levels. According to this guideline, athletes must progress sequentially through each competition tier in order to qualify for higher-level events (7). This ensures a standardized and merit-based pathway for advancement within the Special Olympics competition system.

### 1.3 Research perspectives on unified sports® competitions

The current state of research on Special Olympics Unified Sports® remains underdeveloped, both in general and with regard to competitive contexts specifically (1). Most existing studies focus on the psychosocial outcomes of sports participation for people with and without disabilities, such as socio-emotional learning, personal development, social interaction, and the broader integration of individuals with intellectual disabilities outside of competitive environments (5, 10–12).

Empirical findings related to competition suggest that Unified Sports® often do not facilitate equal conditions between athletes (individuals with intellectual disabilities) and partners (those without disabilities) (1, 13, 14). Asymmetric team structures based on disability status have been observed, with partners frequently adopting supportive roles, both emotionally and in sport-specific tasks. These roles are typically oriented toward fostering the development of athletes, highlighting a tension between social support and the pursuit of athletic success.

One recurring phenomenon is that partners may intentionally reduce their performance to enable athletes to achieve positive experiences (1). Conversely, in some cases—such as in Unified basketball—partners have been found to dominate gameplay, exhibit higher performance levels, and engage more actively than athletes (14). These tensions are compounded by the importance of *togetherness*, a central value for both athletes and partners in Unified Sports® (5).

Despite these insights, the mechanisms underlying such dynamics remain unclear. It is yet to be determined whether these phenomena stem from the Unified Sports® model itself, the characteristics of specific sports, or the nature of joint physical activity between individuals with and without intellectual disabilities.

## 2 Methods

To address the identified research gap, a total of 41 interviews were conducted with players (14 athletes and 27 partners) (see Table 1) who participated in handball and basketball competitions at the 2022 Special Olympics National Games in Berlin. This event can be interpreted as the German national championship in these respective sports within the Unified Sports® framework. Furthermore, the competition served as a qualification event for the 2023 Special Olympics World Games.

**Table 1:** Overview of interviewees.

Players of the Unified Teams	Basketball (7 Teams)		Handball (6 Teams)	
Partners	1 female	12 male	7 female	7 male
Athletes	0 female	3 male	2 female	9 male

The interviewees were drawn from six handball teams and seven basketball teams. Prior to data collection, all teams were informed that a scientific study would be conducted during the competitions. The data collection procedures were communicated in a transparent manner. All participants received information regarding data protection regulations and ethical standards in accordance with established research guidelines. Potential interview participants were identified through a purposive sampling approach, whereby team coaches were asked to inform and invite athletes and partners who expressed an interest in participating in the study. Participation was entirely voluntary.

The semi-structured interview guide began with an open-ended narrative prompt inviting participants to reflect on their own team and personal sports history in handball. Subsequent questions focused on the participants' individual role within the team, experiences during the competition at the National Games, and perceptions of the scouting and qualification processes for the following year's World Games.

All interviews were transcribed verbatim and analyzed using the methodology of Grounded Theory [GTM; (15)], following procedures of open and axial coding. The analysis was supported using the qualitative data analysis software MAXQDA. The coding process was conducted collaboratively by the research team, and emerging findings were discussed jointly to ensure consistency and analytical depth. In line with GTM's iterative approach, this analytical process remains ongoing at the current stage of the study.

The phenomena and action patterns presented in the results section will be further refined through an additional round of data collection, expanded axial coding, and—if warranted—selective coding. The primary objective at this stage is to develop a robust and differentiated category system. Whether the final stage of selective coding will be both feasible and necessary cannot be conclusively determined at this point in the research process.

## 3 Results

In reconstructing the players' perspectives on the Unified competition, several distinct areas of phenomena emerged, which are presented in the following section. At the current stage of the study, final categories have not yet been fully developed. Therefore, the naming of the identified (areas of) phenomena was conducted using alternative approaches, such as *in vivo* codes or descriptive labels.

### 3.1 Unclear goal orientation for the National Games

The interviewed players expressed highly diverse perspectives regarding their objectives for participating in the National Games. Particularly among the Unified partners, considerable ambivalence and role ambiguity became apparent. This is illustrated by the example of Unified Partner 6, who stated:

“I would be really happy if my athletes—no, it's not about me—but if my athletes had something they could take home and show their friends. You know, that they are proud they won a medal. Whether it's bronze, silver, or gold, doesn't matter.” (UP6, Pos. 58).

Here, the partner reflects on his role within the Unified team. While he clearly desires sporting success for the team, he explicitly distances himself from personal ambitions and instead aims to facilitate a meaningful experience for the athletes. He assumes that the athletes value competitive success and positions himself as a supporter in achieving this goal. This aligns with previous research findings that emphasize the supportive function of partners within Unified Sports® teams (1).

However, a later statement from the same interview reveals a contrasting emphasis:

“( … ) generally, in the grand scheme of things, it's really all about fun, about being part of it; you know, we came here with a huge group, and the most important thing is that we all have fun together.” (UP6, Pos. 66).

In this statement, the respondent de-emphasizes the competitive outcome and instead foregrounds enjoyment and shared group experience as the highest priorities. These two interview excerpts highlight a clear ambivalence regarding goal orientation within the partners. The partner does not appear to pursue athletic success for himself, but rather considers his contribution successful when the athletes experience success. This suggests a specific approach to gameplay among partners, which will be further explored in a different phenomenon.

The ambivalence evident in the partners' statements is also reflected in the interviews with the athletes. This is illustrated by the example of Athlete 2:

“And of course, primarily to have fun, but winning is also nice.” (UA2, Pos. 70).

The athlete emphasizes fun as the primary motivation. At the same time, however, the importance of winning is also highlighted. This raises the question of which priority truly takes precedence, leaving the underlying goal orientation ambiguous.

The analyses of the interviews suggest that the goal of winning competitions—typically associated with competitive sport—retains relevance within the context under investigation. However, this objective is not equally emphasized by all participants. Notably, the Unified partners exhibit a degree of ambiguity, often projecting competitive aspirations onto the athletes rather than identifying with them personally. This stance may reflect a form of paternalism and, by extension, indicate an underlying hierarchical structure within the team.

### 3.2 Putting athletes in position

A recurring theme across the interviews with Unified Partners is their clear orientation toward a supportive role during gameplay. Their self-conception is closely tied to enabling the participation and positive experience of the athletes. This understanding is exemplified in the statement of Unified Partner 14, who explicitly distances himself from a central or dominant role on the field:

“I really see my role as a supporter and not as someone who is supposed to be in the spotlight here” (UP14, Pos. 101).

This quote indicates a role orientation that prioritizes the athletes' visibility and autonomy over personal performance. The Unified Partner emphasizes the importance of stepping back to create space for the athletes to take center stage.

Unified Partner 4 articulates this positioning with even greater specificity. In the following quote, she reflects on the deliberate choice to hold back during potentially game-deciding moments:

“( … ) of course we can go for the goal ourselves; but it's important that they are in the foreground; and that they have fun; we too of course but it's their thing; and their matter; and they should enjoy it.” (UP4, Pos. 32).

This statement reveals a strong orientation toward the athletes' subjective experience and emotional engagement with the game. The repeated use of “their” underscores a perceived ownership of the sporting event by the athletes. In line with this, the partner describes a strategy of self-limitation: choosing not to take shots on goal themselves, even when the opportunity arises, in order to facilitate athlete action and agency. And this statement illustrates not only the supportive attitude of the partner but also the underlying hierarchy based on ability status.

Such practices illustrate how Unified Partners actively shape the interactional space on the field. Their role is not merely reactive or passive but involves intentional actions aimed at fostering participation, autonomy, and a meaningful sports experience for the athletes. This reflects a deeply internalized ethic of support that permeates both verbal self-descriptions and reported game behavior.

### 3.3 Letting the opponent play

The supportive orientation described by the Unified Partners becomes particularly apparent in interactions with opposing teams. Notably, the goal is not to dominate or dismantle the opponent's play. On the contrary, there is an expressed intention to also enable a positive game experience for the opposing team. This attitude is clearly articulated by Unified Partner 13:

“And then we think to ourselves okay, we could just take out your best player, we could put one of our partners on him, and then he won't do anything anymore. But what's the point of that? Your flow of play is broken. And then you just have to run around in front of our defense. So, we did talk about this once, but we thought, that's not the idea of Unified. That we take away @the athletes@ .… ” (UP13, Pos. 62).

In this sequence, the partner describes a game situation in which a deliberately less skilled player from their own team was assigned to defend a key player from the opposing team. The aim of this tactical decision was to allow the opposing team to maintain their rhythm and flow of play. Once again, the guiding principle is to ensure that the Unified athletes have a meaningful and enjoyable game experience. Notably, this consideration extends beyond the athletes on one's own team to include those on the opposing side. This statement illustrates again the supportive attitude of the partner and also the underlying hierarchy based on ability status.

Such deliberate restraint and strategic consideration for the opponent are highly atypical within conventional competitive sports. However, within the context of Special Olympics competitions, this appears to represent a distinctive feature of gameplay dynamics. It reflects an alternative value orientation—one that prioritizes mutual respect, participation of athletes, and the co-construction of a positive sporting experience over traditional notions of competition and dominance.

## 4 Discussion

The phenomena described point to pronounced hierarchies within Unified Sports® teams, which appear to be closely linked to the category of disability. These hierarchies are further reinforced by the competitive context itself. Despite the formal regulations of the Competitive Model (7), which explicitly call for equitable performance and the active involvement of all team members, the data reveal that Unified Partners frequently adopt a reserved, supportive role (1).

A central pattern is the intentional withdrawal of partners from game-deciding situations in order to place athletes at the center of play. This posture extends beyond intra-team dynamics and can also be observed in interactions with opposing teams. For example, some partners deliberately avoid exploiting their athletic superiority in order to preserve the opponent's rhythm and ensure an enjoyable experience for all participants. In these instances, the emphasis shifts from winning at all costs to co-creating a meaningful sporting encounter. Similarly, shared enjoyment within the team is frequently prioritized over competitive success. This appears to be a key condition for the success of Unified Sports® competitions. At the same time, partners assume that winning remains important for the athletes themselves. The phenomena described warrant more nuanced theoretical analysis and conceptual framing in future research. This is particularly pertinent given the occasionally paternalistic behavior of the partners, which calls for critical reflection, as such practices may undermine core principles of competitive sport and challenge conventional logics of athletic performance.

This ambivalence reflects a fundamental tension between the formal logic of competition and the lived practices observed at Special Olympics events. The principle of *Meaningful Involvement* is interpreted in markedly different ways—ranging from active participation in sport-specific actions to social inclusion and emotional engagement (8). Such variation introduces the risk that well-intentioned support may slip into paternalism, ultimately constraining athletes' autonomy and sense of self-efficacy (1).

The practices observed challenge conventional understandings of competition (6). Rather than being dismissed as deviations from “real sport” they may be understood as alternative and equally valid forms of athletic engagement—forms grounded in solidarity, participation, and mutual recognition. These findings underscore the need to broaden normative frameworks of competition to better account for values of equal participation and relational dynamics within Unified Sports®.

### 4.1 Future directions

As the study is grounded in the research paradigm of Grounded Theory Methodology, a second phase of data collection has been initiated. In this phase, basketball and handball teams were observed and studied during the Special Olympics World Games 2023. In addition, floorball teams were accompanied and researched during the 2024 National Games and the upcoming 2025 World Games. These new data sets will be integrated with the previously collected material to generate a clearer and more differentiated understanding of the emerging phenomena. In the continued presentation of the results, it will be important to place greater emphasis on the experiences of the partners. This perspective has been comparatively underrepresented in the current report and warrants further analytical consideration.

At the theoretical level, and in light of the empirical findings, it appears essential to critically examine the concept of inclusion as it is employed in the official documents of Special Olympics. Likewise, the notion of paternalism (16) should be explored and contextualized more thoroughly. Similarly, the concept of relational autonomy (17) provides a theoretical lens through which the dynamics between participants with and without disabilities can be reinterpreted, challenging individualistic assumptions about agency and interdependence. Clarifying these concepts may support a more nuanced and in-depth interpretation of the statements made by partners—and, in the future, also by coaches.

This extended data set will also allow for a comparative analysis between the different sports. Potential differences between sports may emerge and will be systematically explored. In addition, the perspectives of coaches, referees, and observers will be examined to gain an even more comprehensive understanding of Unified competition. In order to capture the different areas of the research field as precisely and comprehensively as possible, the various sports and actor groups were initially analyzed separately. This separate analysis allowed for a more nuanced understanding of context-specific dynamics and perspectives. Based on these individual analyses, comparative approaches were subsequently applied, with the form of presentation chosen in each case aiming to highlight the most in-depth and meaningful insights emerging from the data.

The results obtained thus far cannot yet be considered valid, as the identified phenomena still show substantial overlap and lack analytical distinctiveness. This limitation further underscores the need for continued data collection.

Following this extended data collection, a comprehensive analysis of all material will be conducted. The newly collected data will be subjected to open coding, after which all data sets—both new and previously collected—will be analyzed through axial coding. The aim of this process is to develop a robust and final category system. In this context, a decision will also be made as to whether the final step of selective coding is both possible and necessary.

## 5 Conclusion

This study sheds light on the complexities of competitive dynamics in Unified Sports®, revealing how relational structures and values centered on equal participation challenge traditional competition models. While provisional, these insights offer a promising foundation for rethinking athletic engagement that emphasizes mutual respect and shared experience.

## Data Availability

The original contributions presented in the study are included in the article/Supplementary Material, further inquiries can be directed to the corresponding author.
